# Situational analysis of hypertension management at primary health care level in São Paulo, Brazil: population, healthcare professional and health system perspectives

**DOI:** 10.1186/s12913-024-10978-1

**Published:** 2024-05-28

**Authors:** Marta S. Palmeirim, Yara C. Baxter, Mariana Silveira, Rafael V. Maggion, Beatriz Aquino, Álvaro Avezum, Jasmina Saric, Louise Morgan, Luciano F. Drager, Luiz A. Bortolotto, Suely Miya S. Rollemberg, Marcia M. C. de Lima, Edmir P. R. Albuquerque, Olivia Jones, Peter Steinmann, Theresa Reiker, Johannes Boch

**Affiliations:** 1 Swiss Tropical and Public Health Institute, Allschwil, Switzerland; 2 University of Basel, Basel, Switzerland; 3 Novartis Foundation, Basel, Switzerland; 4 Instituto Tellus, São Paulo, Brazil; 5 Sociedade de Cardiologia do Estado de São Paulo (SOCESP), São Paulo, Brazil; 6 Hospital Alemão Oswaldo Cruz, São Paulo, Brazil; 7 São Paulo University, São Paulo, Brazil; 8 McMaster University, Hamilton, Canada; 9 World Heart Federation, Geneva, Switzerland; 10 American Heart Association, Dallas, TX US; 11 Unidade de Hipertensão, Instituto do Coração (InCor), Hospital das Clínicas, University of São Paulo, São Paulo, Brazil; 12 Sociedade Brasileira de Hipertensão, São Paulo, Brazil; 13 Secretaria Municipal da Saúde, São Paulo, Brazil

**Keywords:** Hypertension, Cardiovascular diseases, Situational analysis, Primary health care, Brazil, Design thinking

## Abstract

**Background:**

Government-led, population-wide initiatives are crucial for advancing the management of hypertension − a leading cause of cardiovascular disease (CVD) morbidity and mortality. An urban population health initiative was conducted against this backdrop, focussing on hypertension in the primary health system in São Paulo, Brazil. Within the frame of the initiative and under the supervision and leadership of the municipal health authorities, a situational analysis was conducted on the needs in hypertension management, marking the first phase of a Design Thinking process. This article describes the situational analysis process and presents the identified elements to be strengthened considering hypertension diagnosis, treatment and control.

**Methods:**

First, a mixed-methods approach was used, starting with a literature review of municipal hypertension data followed by meetings (*N* = 20) with the local public health administration to assess health system level components. To investigate activities on hypertension diagnosis, treatment and control, nine primary healthcare units were selected from two districts of São Paulo city– Itaquera and Penha– which received an online form addressed to managers, participated in conversation circles of staff and patients, and underwent shadowing of community health agents.

**Results:**

Data gave rise to two main outputs: (i) a patient care journey map; and (ii) a matrix summarizing the identified needs at patient, healthcare professional and health system level for diagnosis, treatment and control of hypertension. Patient awareness and knowledge of hypertension was found to be insufficient and its management needs to be improved. For health professionals, disease awareness, technical training, more time dedicated to patients, and simplified guidelines and clinical decision-making tools for hypertension management were identified as principal needs. The situational analysis found that the healthcare systems efficiency might be improved by establishing defined treatment and care delivery goals with a focus on outcomes and implemented through action plans.

**Conclusions:**

This situational analysis identified several needs related to hypertension control in São Paulo that are in line with global challenges to improve the control of CVD risk factors. Findings were also confirmed locally in an expansion phase of this situational analysis to additional primary care facilities. As a consequence, solutions were designed, promptly taken up and implemented by the municipal health secretariat.

**Supplementary Information:**

The online version contains supplementary material available at 10.1186/s12913-024-10978-1.

## Background

Hypertension is the leading cause of death globally. The condition affects about 1.4 billion adults and is the main risk factor for cardiovascular diseases (CVD) [1, 2]. The management of hypertension improves cardiovascular population health, reduces early mortality and avoidable hospitalizations [2–4]. 70% of people with hypertension live in low- and middle-income countries, where health systems are largely focused on the delivery of services for acute healthcare needs, as opposed to managing chronic conditions amenable to prevention. The latter requires long-term sustained engagement and interaction between patients, healthcare professionals and the health system to efficiently manage conditions [5], improvement in event-free survival along with better quality of life, and medical cost reduction. To achieve these goals, the identification of specific barriers and needs related to effective hypertension control is required to develop targeted interventions and spark the necessary transformation of health systems and their services delivery models [6].

Like many other countries across the globe, Brazil has undergone a major shift in its epidemiological profile; burdened historically by infectious diseases, the country’s main public health challenges today stem from non-communicable diseases (NCDs) [7]. The increase of NCDs observed in Brazil and elsewhere was precipitated by an increasingly sedentary lifestyle and changing eating habits, leading to a high prevalence of obesity and other CVD risk factors [2, 8]. In 2016, CVDs were the leading cause of morbidity and mortality [9]. A recent study exploring hypertension prevalence in different South American countries found a 53% prevalence among 5,557 Brazilians aged 35 to 70 years from both rural and urban settings [10]. Investing in corrective and preventive behavioural approaches in the face of ever-increasing treatment expenses has become a priority for the government [11, 12]. Studies have shown that the Brazilian primary health system should be strengthened in the area of disease management pathways (including for NCDs) with a people centred perspective [13].

The challenges of NCDs, as in other parts of the world, also impact São Paulo city, Brazil’s most populated city, despite the constant efforts of local health authorities to reduce their prevalence and burden [14, 15]. In São Paulo, the prevalence of hypertension in the adult population has been estimated to be 23.2% in a telephone-based survey [16]. However, the same survey found large variability in prevalence within the adult population; in people aged 50 to 69 years of age, the prevalence of hypertension varied from 34.5 to 51.3%. In São Paulo city, half of the population depends on Brazil’s unified public health system (Sistema Único de Saúde; SUS), which, among other services, provides universal coverage and access to free medication [17]. Through different initiatives and reforms, the public health program for São Paulo focuses on the reduction of mortality due to NCDs and includes hypertension management. Prominent examples are the NCD Strategic Action Plan to which São Paulo state adhered to, the tobacco control laws, salt reduction policies and public sport facility programs, which all have relevance for municipal planning of priorities [18, 19]. A study from 2011, investigating care pathways of already diagnosed hypertensive patients in the public health care sector of São Paulo, documented a treatment adherence level of 20% [20] and that 35–50% of patients treated for hypertension had their blood pressure controlled [20, 21]. These findings reveal opportunities to increase treatment adherence, quality of care, access to medicines, patient follow-up and care coordination [14].

The initiative Better Hearts Better Cities (entitled “Cuidando de Todos” in Brazil), applying the CARDIO (Care, Access, policy Reform, Data and digital, Intersectoral collaboration and local Ownership) approach [22], was designed as a multidisciplinary, multisector initiative to support local authorities in addressing urban cardiovascular population health, including hypertension as primary risk factor. The initiative was implemented in Ulaanbaatar, Mongolia; Dakar, Senegal; and São Paulo, Brazil. In Brazil, the initiative was executed in collaboration with the São Paulo Municipal Health Secretariat (Secretaria Municipal de Saúde; SMS-SP). This partnership aimed at co-creating and accelerating the implementation of a model to approach NCDs. The implementation methodology was guided by a Design Thinking approach [23]. The current article details the diagnosis step of the methodology, which focused on assessing the primary needs in the public health system in São Paulo. The identified needs of patients, healthcare professionals and the health system with respect to hypertension diagnosis, treatment and control will be presented.

## Methods

### Setting

São Paulo city is one of the five most populous metropolis in the American continent and the largest financial and corporate centre in Latin America [24]. São Paulo was recently included in the “Global Cities of the Future 2021/2022”, occupying the fourth place of the ten “Megacities of the Future” with the best strategies for attracting foreign direct investment [25]. With more than 12 million inhabitants on 1,521 km^2^ [26], São Paulo was selected for the implementation of Better Hearts Better Cities. Reasons include the range of opportunities to improve hypertension and CV risk factor control, São Paulo’s urban characteristics such as the close co-existence of high-, low- and middle-income features, the developed health system (SUS) and the complexity of urban management, especially in health [27]. After agreeing to jointly execute the initiative, the SMS-SP selected the districts of Itaquera and Penha to host the first implementation.

### Conceptual approach to problem identification

In São Paulo, the initiative was implemented between 2018 and 2020 in the stages of groundwork, diagnosis, exploration, co-creation, implementation and scale-up. This initiative followed a Design Thinking process [23], using the double diamond approach [28]. Besides factual roadblocks and concrete challenges, the Design Thinking process also considers latent needs of the health system. These needs may be abstract and emotional and, as such, can be difficult to express or to recognize in traditional needs assessments [29]. To address this, Design Thinking focuses on the analysis of the end-user and beneficiary perspective in a clarification step (here referred to as situational analysis). Information collected in this step through interviews and shadowing is essential to frame key issues correctly, identify opportunities for improvement and ideate adequate solutions [30], which can be subsequently implemented in partnership with the local health authorities. This situational analysis and its results of the health care ecosystem’s needs for patients, healthcare professionals and the system itself are described in the current manuscript. The process used to conduct this situational analysis is aligned with the recommendations of the World Health Organization (WHO) HEARTS package [31] and the World Heart Federation [6].

### Primary health care structure in São Paulo

The situational analysis took place in 2018 in the district of Itaquera and in 2020 in the district of Penha. These districts were selected due to their representativeness of low- and middle-income communities. Additionally, primary healthcare units (Unidades Básica de Saúde; UBS) with different health system management models are present in these districts; the primary health care network of São Paulo is composed of 450 UBSs (traditional model) [32] and 117 outpatient medical care centres (Assistência Médica Ambulatorial; AMA) [33], of which 87 are integrated with a UBS [34]. The UBS network is staffed by different types of primary care teams (Equipes de Atenção Básica; EAB), including those organized according to the family health strategy (Estratégia Saúde da Família; ESF), a Brazilian management model of primary care delivery [35]. In Brazil, the organization, management and execution of services and activities of primary health care is the responsibility of the municipality. In São Paulo, health facilities can be directly managed by the SMS-SP (direct administration) [36, 37] or, alternatively, the management can be delegated to social organizations (e.g. cooperatives), private companies, non-governmental or philanthropic organizations [38]. Table 1 depicts the characteristics related to primary care infrastructure in Itaquera, Penha and São Paulo city as a whole.

**Table 1:** Characteristics of São Paulo and the two participating districts, Itaquera and Penha. Data extracted from TabNet São Paulo of the SMS

	Total population	Mortality from ischemic heart disease (per 100’000)	Number of UBSs				Total number of health staff	Other secondary / tertiary healthcare services
			AMA/UBS	ESF	Traditional UBS	Total		
São Paulo	11’753’659	68.2	53	300	116	469	NA	556
Itaquera	549’220	81.7	7	10	7	24	2’051	20
Penha	473’549	78.2	4	7	10	21	1’393	21

AMA/UBS, Assistência Médica Ambulatorial com Unidade Básica de Saúde integrada (outpatient medical care center integrated with a primary healthcare unit (UBS, Unidade Básica de Saúde)); ESF, Estratégia Saúde da Família (primary healthcare unit with family health strategy); LMI, low- and middle-income; NA, not available

### Situational analysis procedures

The situational analysis was conducted by a team composed of members of the Novartis Foundation (global initiative coordinator), the Instituto Tellus (local implementation partner), the São Paulo Society of Cardiology (Sociedade de Cardiologia da Cidade de São Paulo; SOCESP), as well as members of the SMS-SP. The municipal authorities approved the initiative and its strategy, and took ownership for the execution, facilitated communication with internal stakeholders, helped establish a first technical working group and identified the first implementation districts. The SMS-SP assigned the initiative to the chronic diseases and NCD care (Doenças e Agravos Não Transmissíveis; DANT) technical department. This department was created in 2018 to elaborate a municipal care protocol for NCDs and harmonize the use of different national and international guidelines by physicians in the public health system of São Paulo. Thus, the initiative fully supported the priorities of the SMS-SP.

With this governance established, the team started the first part of the situational analysis by addressing health system components with a focus on primary care delivery. The investigation was conducted at the SMS-SP, in order to understand health system building blocks, such as governance, importance of and priority given to primary care, currently available resources and gaps, data management priorities and available information management systems, current guidelines, their application, and the policy landscape. Special consideration was on other programs related to hypertension in Brazil, and recent initiatives to address other chronic diseases such as diabetes. To complement this information, the team conducted a review of the available literature and municipal data concerning CVDs for São Paulo.

Secondly, continuous follow-up discussions with the NCD technical department at the SMS-SP supported the contextualisation of the findings from the first part of the analysis. Inter-disciplinary workshops with relevant partners (e.g. SMS-SP, public officials representing primary care and/or NCD management, district health care delivery, medical societies, global initiative coordinator, local implementation partner, international scientific partners) were conducted to further deepen the understanding of the health system concerning diagnosis, treatment and control of hypertension. The discussions clarified the relation of municipal strategy to operational execution in the UBS in terms of technical decisions, perception and execution of best practices.

Finally, a total of nine UBS (six in the district of Itaquera and three in the district of Penha) were selected by the SMS-SP to be included in this situational analysis and undergo a thorough investigation using several data collection tools.

### Data collection approaches and tools

To gain deep knowledge and understanding of the entirety of the health system, a mixed-methods approach based on the Design Thinking process [23, 39] and the AEIOU framework (Activities, Environments, Interactions, Objects, and Users) was pursued [40]. In addition to the use of these frameworks, to gain a complete understanding of the conditions and potential for interaction with the population in and beyond the health system, other than primary health care unit workers, the team also included relevant focal points within the district, such as community champions (e.g. samba and football club, spiritual leaders) and reference points (e.g. shopping mall, high traffic venues, green areas). Table 2 presents the type of information collected from the different stakeholder groups to help pinpoint unmet needs at primary care level. This was executed using three tools targeting different respondents based on their role in the health system:

1. An online form consisting of open- and closed-ended questions was applied to collect data for an initial diagnosis of the needs in each UBS (Supplementary files 1 and 2; one for Itaquera and one for Penha). An online version of the online form was sent to the UBS managers who were invited to complete it. The online form covered, among other topics, physical and personnel structure of the UBS, use of digital technology, data collection and recording tools and service time of each professional. Additionally, there were a few questions, targeting the work conducted by the community health agents (CHAs), regarding their knowledge around hypertension, their degree of patient engagement, their knowledge on use of medication, and their practice in encouraging healthy lifestyle behaviour. Between the implementation of the online form in Itaquera and Penha, it underwent some adaptations and fine-tuning based on the experience in Itaquera; hence, there were two versions of the online form (Supplementary files 1 and 2).
2. Conversation circles (*n* = 43) were carried out with UBS staff and patients in the UBS offices. For each respondent category, a specific conversation guide with lead questions was available. The goal was to, in each UBS, include one person from each of the following categories: (i) patient; (ii) doctor; (iii) nurse; (iv) community health agent; (v) pharmacist; (vi) admission officer; (vii) UBS manager, and (viii) school health programme manager. Participants were from the same district and selected by the UBS. Additionally, the situational analysis team included experts in the area of cardiovascular health, population health and health systems, among which were directors of medical societies such as SOCESP, NCDs forum [41] and representatives of the Brazilian Ministry of Health.
3. Shadowing was conducted by members of the implementation team who observed two CHAs from one selected UBS throughout their day of work [40]. The main goal of this approach was to understand the relationship between CHAs and hypertensive patients. Additionally, CHAs were asked about their beliefs, attitudes and perception of hypertension, as well as about their observations and perceptions on the population’s diet and physical activity in the context of the disease cluster. Observations and responses were recorded using a standard data collection form (Supplementary file 3).

**Table 2:** Main topics investigated in the situational analysis and from whom the information was collected through online forms, key informant conversation circles and/or shadowing

Stakeholder	Investigated topics
Front (direct contact with patients)	
UBS managers	- Primary care management - Challenges in primary care - UBS’s challenges related to hypertension - UBS’s goals and indicators related to hypertension
Doctors (i.e. general practitioner, paediatrician, gynaecologist)	- Routine medical care protocol - Medical protocol for hypertensive patients - Criteria for referral to a specialist - Medication used for hypertension and dosage/frequency
Nurses	- Routine care protocol of nurses - Protocol of care of nurses for hypertensive patients
Nursing assistants	- Routine care protocol of nursing assistants - Protocol of care of nursing assistants for hypertensive patients
Pharmacists and pharmacy technicians	- Routine pharmaceutical care protocol - Pharmaceutical care protocol for hypertensive patients
Front desk staff	- Daily reception service protocol - Reception care protocol for patients with hypertension
NASF professionals (physiotherapists, speech therapists, nutritionists, physical educators)*	- Care protocol for NASF/ESF professionals in everyday life - Care protocol for NASF/ESF professionals for patients with hypertension
Family and community doctors*	- Routine family and community medical care protocol - Family and community medical care protocol for hypertensive patients - Particular characteristics of a family and community doctor’s work
Community health agents*	- Routine care protocol of community health agents - Protocol of care of community health agents for hypertensive patients
Patients	- Overall perception of the service of UBSs - Treatment and follow-up of hypertensive patients - Participation in group activities - Quality and type of interactions with healthcare professionals
Local community leaders (e.g. NGOs, community, church)	- Relationship/interaction with the UBS - Partnership activities between the UBS and the community
Back (e.g. management, monitoring, logistics structuring)	
Responsible for primary care in the SMS	- Role of the Technical Area of Primary Care in the management of CRS/STS/UBS - Main challenges encountered in the interface between the UBS, STS, CRS and SMS
Interlocutor for NCDs in CRS	- Role of the CRS in the management of STS/UBS - Main challenges in the interface between the UBS, STS and CRS
Interlocutor NCDs in STS	- Role of STS in the management of UBS - Main challenges in the interface between the UBS and the STS
Interlocutor NCDs in OSS	- Role of the OSS in the management of UBS? - Main challenges in the management of the UBS by OSS?
Responsible for pharmaceutical programs in the SMS	- Role of pharmacists in the UBS? - Main challenges related to the pharmaceutical assistance in primary care - Main challenges related to hypertension and the pharmaceutical assistance
Responsible for the system in the SMS	- Existing indicators and goals for hypertension and their monitoring processes - Existing databases and their formats
Medical societies and other organizations	- Sharing of good practices and existing protocols related to hypertension

CRS, Coordenadoria Geral de Saúde (regional health coordinator); ESF, Estratégia Saúde da Família (family health strategy); N/A, not applicable; NASF, Núcleo Ampliado de Saúde da Família (expanded family health center); NCDs, non-communicable diseases; NGO, non-governmental organization; OSS, Organização Social de Sáude (health social organization); SMS, Secretaria Municipal de Saúde (municipal health secretariat); STS, Supervisão Técnica de Saúde (technical health supervision); UBS, unidade básica de saúde (primary healthcare unit). *Only exist in UBS with NASF/ESF; NASF transitioned into ESF in 2019/2020

### Data analysis

The information gathered by the implementation team using online forms, conversation circles, shadowing activities and government workshops, was compiled, tabulated and systematized by Instituto Tellus. This provided a first assessment of the existing needs, which resulted in two main outputs. The first was a patient care journey map, investigating the interactions between patients and the primary care team and mapping challenges and opportunities. This map served as an interactive and iterative tool during data collection, allowing for the visualization of details of the system through which patients with hypertension navigate and, therefore, helped clarify needs and bottlenecks present at different stages of the patient’s journey. The second main output was a matrix presenting the identified needs presented along three major components (i.e. patients, healthcare professionals and health system) for the diagnosis, treatment and control stages of hypertensive patients.

The gaps identified during this step were taken up in the co-creation sessions with the city health authorities, members of the American Heart Association (AHA), SOCESP and other primary care practitioners for refinement and to brainstorm for solutions. This process has been described in detail elsewhere [23]. All results were discussed and validated by the SMS-SP, which confirmed the adequateness and provided further input.

## Results

All nine UBS managers who were asked to respond to the online form returned it and all 30 people who were planned to include in conversation circles attended.

The nine pilot UBSs were representative of the different UBS administrative models of the Municipality of São Paulo: two traditional UBS, four AMA integrated with a UBSs, and three UBSs with ESF. Of these, one of the traditional UBS was under direct administration and the remaining eight were managed by a social organization. These UBSs served between 12’000 and 39’964 people. The operational models, management types, number of people and adults covered and years in operation for each of the nine UBSs are presented in Table 3. The newest UBS had been in operation for 10 years, whereas the oldest had been established 45 years ago. In terms of data collection systems, all UBS used SIGA Saúde (Sistema Integrado de Gestão de Assistência à Saúde de São Paulo) for the registration of patients’ appointments and to record basic data, and Gestão de Sistemas de Saúde (GSS; Health system Management), which is the medicines logistics system. It was also found that all nine UBSs had an integrated pharmacy.

The analysis of the results from the nine pilot UBSs revealed that, to improve the quality of the data collected, the online form needed to be revised. Questions that were found to not bring relevant insights were removed, (e.g. “how many functioning printers does your UBS have?”), while additional questions were introduced to fill data gaps noticed.

**Table 3:** Overview of characteristics of investigated primary care units

District	UBS number	Operational model	Management type	Number of people covered	Number of adults covered	Years in operation
Itaquera	1	AMA/UBS	OS	19’577	13’723	37
	2	AMA/UBS	OS	26’863	18’831	34
	3	AMA/UBS	OS	18’310	12’835	37
	4	ESF	OS	12’000	8’412	23
	5	ESF	OS	17’370	12’176	33
	6	Traditional UBS	DA	26’412	18’515	45
Penha	7	AMA/UBS	OS	34’936	24’490	12
	8	Traditional UBS	OS	20’110	14’097	38
	9	ESF	OS	39’964	28’015	10

AMA/UBS, outpatient medical care center (Assistência Médica Ambulatorial) integrated with a primary healthcare unit (UBS, Unidade Básica de Saúde); DA, direct administration (goverment; administração direta); ESF, primary healthcare unit with family health strategy (Estratégia Saúde da Família); OS, social organization (organização social); traditional UBS, traditional primary healthcare unit (UBS)

### Patient care journey map

The results from the online forms, conversation circles and shadowing of CHAs were consolidated and mapped out in the patient care journey map (Supplementary file 4: Figure 1), which catalogues the structure of the observations of primary needs along a timeline. The starting point of each overlapping journey was a research-based fictional persona that represented a particular group (i.e. patient, community health agent, doctor, pharmacist, nurse) [42]. Each persona’s journey started with a short description of the official requirements from the Brazilian comprehensive health and nutrition promotion practices in primary healthcare (Práticas Integrais de Promoção da Saúde e Nutrição na Atenção Básica; PINAB). The journey also showed explicit and latent needs. By visually exposing the information collected, the team was able to trigger discussions among health managers, clinical team members and health authorities and to understand whether the results obtained by the implementation team corresponded to the actual needs experienced by healthcare professionals and managers. This map helped identify several opportunities on how to improve the patients’ experience. Those participating in the exercise reported that it was the first time this complex system of interactions was depicted in a visual way and was, thus, insightful to gain oversight of paint points, process overlaps and opportunities to optimize team work.

### Main needs and barriers identified

The mapped barriers for the different CVD management stages (i.e. diagnosis, treatment and control) for patients, healthcare professionals and the health system resulting from the pilot phase are presented in Table 4. Conversation circles found, for instance, that the patients’ knowledge and awareness of the importance of hypertension risks and of regularly measuring blood pressure was poor, particularly in men and young people. According to patients, these issues, coupled with a poor understanding of the treatment and problems in the health system such as insufficient staff, in turn, according to health professionals, led to low adherence to treatment. From the patient perspective, for successful control of their blood pressure, they needed a stronger bond and a good relationship with the health staff to increase their engagement.

From the perspective of healthcare professionals, there was a lack of training, and the quality in medical record entries required attention. Concerning treatment, healthcare professionals felt that the training of CHAs on the topic of NCDs, the motivation provided to patients and the quality of guidelines needs to improve. As an example, hypertension was diagnosed following the 7th Brazilian guideline [43], but the situation analysis revealed that due to the complexity of the guideline, those best-practices are only partially applied. Concerning the control of hypertension, the presented findings showed that healthcare professionals needed more time with patients to explain treatment in detail, but also highlighted some of the inefficiencies in the health system that would need to be resolved.

Concerning the health system, a perception that 15-minute appointments were too short and that this negatively impacted both the diagnosis and treatment steps was mentioned by both health professionals and patients. Finally, the system also lacked clear goals and action plans when it came to diagnosis, treatment, and control (i.e. follow-up of patients).

For each of the needs and barriers identified for patients, health professionals and the health system (Table 4), brainstorming sessions led to the outlining of prototype solutions. These prototype solutions later underwent a selection phase and were developed into the solutions that were implemented; these final solutions are presented in a second publication in this series of publications on the Better Hearts Better Cities initiative in Brazil.

**Table 4:** Needs for hypertension services captured by stage and by stakeholder level

	Diagnosis	Treatment	Control	Prototype solutions
Patients	*NCD management*: • Hypertension is not a priority in routine primary care clinic activities • BP is not measured nor recorded regularly, thus, patients do not have any possibility to track their status • As it is an asymptomatic disease, care is sought belatedly • Low number of men and youth seeking care in the UBS	*Low adherence to treatment is associated with*: • Difficulties in primary care service such as insufficient staff, atmosphere, behavior of some collaborators • Missing information on prescription and medication-based or non-medication treatment • Poor understanding of need for treatment leads to discontinuation	*Difficulties to bring condition under control is connected to*: • Low engagement in the care process and consequent impact on control of the disease • Lack of a bond, trust and fidelity between patient and UBS / staff • Difficulties to follow-up adequately	• App for patients to record and track their treatment independently • Hypertension Ambassadors who were responsible for mobilization the local community concerning hypertension for local projects and activities • Inspirational videos with inspiring stories of people from the community who have reference in health care and that the community can identify with. • App directing the patient to the most appropriate public health facility based on his/her symptoms • A card for hypertensive patients to, not only monitor their blood pressure measurements, but also control the follow-up visits and appointments with health professionals at the UBS. • A patient follow-up system in the form of games
Healthcare professionals	*Difficulties in adequately diagnosing are related to*: • Challenges with quality of documentation in patient’s medical record entries • Missing information concerning diagnosis and CVD risk score in medical records • Official courses do not include NCDs and hypertension. (*only PLAMEP courses ensure the discharging of professionals)	*Challenges to achieve control*: • Missing standardization of treatment and follow-up processes • CHA training does not address NCDs • Physicians do not motivate patients to adhere to non-medication treatment • Poor understanding of guidelines by healthcare professionals	*Perceived difficulties on control*: • Missing standardization of treatment and follow-up processes; an overlap in care strategies and protocols cause confusion and inefficiency • Physicians do not have enough time to explain treatment in detail • Some medical cases referred to specialists could have been solved in primary care	• Unique protocol for all healthcare professionals to follow on NCD/CVDs management and training • Management and productivity training for unit managers, based on case discussions • Appointments with specialist healthcare professionals by teleconference • An app used as a risk calculator with decision support to help health professionals to categorize the patient’s risk • Training and supporting material for CHA focused on chronic diseases
Health system	• 15 min is not enough time for a medical appointment to be carried out (especially in the case of comorbidities) • Absence of goals and action plans for active screening	• 15 min is insufficient time for an appointment where proper instructions are provided • Absence of goals and action plan for physicians and patients • Lack of a defined and structure process to distribute tasks and duties in the team • Ineffective communication among health authorities	• Management is only focused on prescription volume, not patient-outcomes • Absence of defined goals in following-up on hypertensive patients (NCDs), until they are “treated and controlled” • UBS with no ESF does not monitor patients by risk criteria	• A scheduling system in which appointments are booked in advance, ensuring that a specific time is available for hypertensive patients • Have educational/informative activities in the waiting room, with the support of the unit’s medical team and external partners • An online form that patients respond to while they are in the waiting room to optimize the time with the health professional and make the appointment more efficient • Support material for the administrative staff referring patients to secondary care • Proposal of qualitative indicators vs. quantitative for social health organizations to define the patients’ status, such as whether they are controlled or not • Reviewing past medical records to do the identification and stratification of the risk of those hypertensive patients which had not yet been done. This could be held on weekends, with support teams. • Developing a BP screening room in the units with proper ambience and signaling for resting and better BP measurement.
	Needs which were identified across the diagnosis, treatment and control: • Missing data and overview on the patient population (number of hypertensive patients with active registration vs. estimated prevalence) • Strategy for patient data collection can be optimized • Different data collection systems • High turn-over of staff • Missing coordination between staff and possibilities to optimize in-clinic coordination			• Keeping a spreadsheet with hypertensive patients’ information and log of their visits for a systematic and active follow-up based on their risk stratification • Create a moment in team meetings to discuss the main cases of patients with hypertension, present metrics and data from the patients’ survey; this could help the team decide which actions could be taken to qualify the care to this type of public

BP, blood pressure; CHA, community health agent; CVD, cardiovascular disease; ESF, Estratégia Saúde da Família (family health strategy); NCD, non-communicable disease; PLAMEP, Plano Municipal de Educação Permanente (municipal permanent education plan), UBS, Unidade Básica de Saúde (primary healthcare unit)

## Discussion

The situational analysis and its findings were the first steppingstone of the Design Thinking process used in the urban population health initiative, Better Hearts Better Cities, in São Paulo city. The data captured on health system components and different stakeholders, such as patients, healthcare professionals, managers and decision-makers, has led to the identification of several needs relating to the diagnosis, treatment and control of hypertension in low- and middle-income settings. Findings were used as a basis for the design of specific interventions targeting the identified needs, which were subsequently taken up and implemented.

The analysis showed that the patient’s perspective encompassed elements connected to disease awareness (specifically regarding men and youth), self-care (including no standard possibility to track disease status, low adherence, missing follow-up medical appointments, and low motivation), difficulties of patients to engage in the care process, build an emotional connection to primary care teams, and lack of consistent early detection and standards of care. For healthcare professionals, needs in optimizing and clarifying guideline-based care, treatment and follow-up, improving information management (e.g. quality of medical record and completeness of documentation), enhancing standard continuous training programs on disease and treatment, and reinforcing team-based care were identified. From the health system perspective, potential was seen to define structured processes to upgrade efficiency, and establish defined treatment and care delivery goals with a focus on outcomes, action plans and applicable guidelines and tools on diagnosis, treatment and control. All the findings of this situational analysis were later confirmed during an expansion phase of the same methodology implemented in another 18 UBS in Itaquera and 18 UBS in Penha. Additionally, the results of this analysis are in line with roadblocks identified by entities such as the World Heart Federation [6] or the Organisation for Economic Co-operation and Development [44]. Hence, it is assumed that the identified challenges including primary care management, patient pathway/journey, and workforce up-skilling are transferable to other geographies and health systems.

To our knowledge, this work is the first that displays the health system in São Paulo or even Brazil, its team structures, responsibilities, and needs on a comprehensive patient journey map. This situational analysis found that, although aware of the complexity of the health system, the visualization of the journey map gave health system managers and healthcare professionals a new perspective on the system that they navigate daily. This provided valuable insights for discussion of opportunities and challenges to address latent and explicit needs, process optimization and task-shifting.

In the context of this urban population health initiative, we evaluated the existing gaps in the healthcare system by engaging with health professionals and patients. This situational analysis provided valuable insights that directly fed into the design of specific interventions and further informed the CARDIO4Cities approach. The CARDIO4Cities approach, which stands for Care, Access, Policy Reform, Data and Digital, Intersectoral Collaboration, and Local Ownership, as described by Aerts and Boufford [22], was developed based on these insights. This comprehensive situational analysis aided in identifying the specific needs and opportunities within the healthcare system, enabling the development of targeted interventions. The implementation of the CARDIO4Cities approach in São Paulo, guided by the São Paulo health authorities, has been proven to be impactful [45, 46]. We believe that a precise understanding of the situation and the healthcare system’s needs is crucial for devising tailored strategies to improve cardiovascular health. During the diagnostic phase, the information gathered through this situational analysis was used to co-create a total of 14 solutions in collaboration with various stakeholders. The objective of these solutions was to reform hypertension management, optimize healthcare delivery, and increase cardiovascular population health.

This process described in the paper showed that despite being resource-intensive due to the assessment being embedded in the primary care context and its daily operation, the methodology led to findings that were perceived to be focused on core needs of the beneficiaries, namely the patients, healthcare professionals, managers and decision-makers and as such were meaningful, endorsed and accepted as appropriate. The findings allowed for the development of sustainable interventions, enabling local stakeholders to feel fully involved and empowered in the whole process. This focus on actual needs is a major strength of the approach, which was reinforced by the bottom-up analysis of the situation. Another strength is that this initiative was able to bring together all types of stakeholders in a participatory and inclusive manner making sure all voices were heard. Finally, the careful selection of the pilot UBSs, whereby every administration model was represented, was of great importance.

Limitations of this work include that the first version of the online form was too complex for respondents and, therefore, it needed to be adapted. Consequently, the composition of questions asked to UBS managers slightly differed between the two districts. Moreover, the team realized, at the start of the project, that some stakeholders felt they might be judged by what they mentioned, feeling observed and showing some discomfort when it came to sharing any bottlenecks in the system. This hesitancy was, most likely, a result of previous negative experiences where the same stakeholders participated in projects without ever seeing any outcomes or benefits. Thus, it was fundamental to clarify that the aim was to build a partnership offering a safe space to share any issues, in a constructive manner. Finally, it was not always easy to obtain consistent data on CVDs from the different stakeholders as there were several sources to collect the data from. Lastly, the rapid staff turnover at the SMS-SP was, at times, limiting the efficacious progress of the analysis.

## Conclusions

Despite all the efforts the SMS-SP has put into reducing the burden of hypertension in São Paulo city, its prevalence remains high and its control remains low. This situational analysis, conducted in close collaboration with SMS-SP, allowed the Better Hearts Better Cities initiative to apply an innovative methodology to identify opportunities for improvement within the health system and established an operational baseline for boosting the diagnosis, treatment, and control of patients with hypertension living in low- and middle-income settings of São Paulo city. In doing so, it laid the foundation for the design of solutions and successful roll-out and expansion of the project. Based on the experience and results from both the pilot and expansion phases, the team recommends the use of this methodology in future studies that aim at identifying needs within a health system based on a well-defined focal area. Employing bottom-up, collaborative, inclusive and needs-focused approaches drives change management, reinforces sustainability and up-take of solutions in the implementation phase.

### Electronic supplementary material

Below is the link to the electronic supplementary material.

Supplementary Material 1: Questionnaire used in the district of Itaquera.

Supplementary Material 2: Questionnaire used in the district of Penha.

Supplementary Material 3: Shadowing of community health agent (CHA) form.

Supplementary Material 4: Figure 1. Patient care journey map with personas representing patients, community health agents, doctors, pharmacists and nurses. BP, blood pressure; CHA, community health agent; CFF, Conselho Federal de Farmácia (federal pharmacy council); CRF, Conselho Regional de Farmácia (regional pharmacy council); ISA, Inquérito de Saúde (health survey); PINAB, Práticas Integrais de Promoção da Saúde e Nutrição na Atenção Básica (comprehensive health and nutrition promotion practices in primary healthcare); SIGA, Sistema Integrado de Gestão de Assistência (integrated assistance management system); SO, health social organization; SVS/MS, Secretaria de Vigilância em Saúde/Ministério da Saúde (health surveillance secretariat/ministry of health); UBS, unidade básica de saúde (primary healthcare unit).

## Data Availability

The datasets used and/or analysed during the situational analysis are available from the corresponding author on reasonable request.

## Abbreviations

AEIOU: Activities, Environments, Interactions, Objects, and Users
AHA: American Heart Association
AMA: Assistência Médica Ambulatorial (outpatient medical care centres)
BP: Blood Pressure
CHA: Community Health Agents
CFF: Conselho Federal de Farmácia (federal pharmacy council)
CRF: Conselho Regional de Farmácia (regional pharmacy council)
CRS: Coordenadoria Geral de Saúde (regional health coordinator)
CVD: Cardiovascular Disease
EAB: Equipes de Atenção Básica (primary care teams)
ESF: Estratégia Saúde da Família (family health strategy)
GSS: Gestão de Sistemas de Saúde (health system management)
ISA: Inquérito de Saúde (health survey)
NASF: Núcleo Ampliado de Saúde da Família (expanded family health center)
NCD: Non-communicable Diseases
NGO: Non-governmental Organization
OSS: Organização Social de Sáude (health social organization)
PINAB: Práticas Integrais de Promoção da Saúde e Nutrição na Atenção Básica (comprehensive health and nutrition promotion practices in primary healthcare)
PLAMEP: Plano Municipal de Educação Permanente (municipal permanent education plan)
SIGA: Sistema Integrado de Gestão de Assistência (integrated assistance management system)
SMS: Secretaria Municipal de Saúde (municipal health secretariat)
SOCESP: Sociedade de Cardiologia do Estado de São Paulo
STS: Supervisão Técnica de Saúde (technical health supervision)
SUS: Sistema Único de Saúde (public health system)
SVS/MS: Secretaria de Vigilância em Saúde/Ministério da Saúde (health surveillance secretariat/ministry of health)
UBS: Unidade Básica de Saúde (primary healthcare units)
WHO: World Health Organization
