# Supplementary material for: Situational analysis of hypertension management at primary health care level in São Paulo, Brazil: population, healthcare professional and health system perspectives
Source: BMC Health Serv Res. 2024 May 28;24:668. doi: 10.1186/s12913-024-10978-1 (PMC11134720; doi:10.1186/s12913-024-10978-1)
Supplement: Supplementary file 1 — Supplementary Material 1: Questionnaire used in the district of Itaquera. [file 12913_2024_10978_MOESM1_ESM.docx]

**Supplementary file 1.** Questionnaire used in the district of Itaquera.

Hello, we are part of Tellus!

We are working together with the City Hall and the East Zone Health Coordinator on a project focused on improving hypertensive health in some primary healthcare units (Unidades Básicas de Saúde, UBS). Your unit is one of those selected for this first moment. You have probably already received our visit, but if not, we will soon be there. :)

At this point of research and information gathering, we felt the need to confirm and understand some basic information about each unit. Thus, we created this questionnaire. We suggest that you answer it as a team, since some questions will need to be confirmed by other people.

This information will be used so that we can better understand the context of each location and, thus, think collectively about the best way to create solutions that are adaptable to your reality).

We are counting on everyone’s information, thank you very much in advance!

Best regards,

Better Hearts Better Cities Project Team - Tellus Agency

*Mandatory questions

1. What is your UBS? *

*Mark only one.*

- Gleba do Pêssego
- Vila Carmosina
- Parada XV
- Santa Terezinha
- Vila Regina
- Itapema
- Conjunto A.E. Carvalho

1. What is/are the national registry of health establishments (Cadastro Nacional de Estabelecimentos de Saúde; CNES) number of the UBS? *

________________________________________________________________________

1. For how long has the UBS existed? *

________________________________________________________________________

1. What management type does the UBS have? *

*Mark only one.*

- Direct
- Social Organization (Santa Marcelina)

1. What type of service does the UBS provide? *

*Mark only one.*

- UBS
- UBS with Family Health Strategy (Estratégia Saúde de Família, ESF)
- UBS with an Outpatient Medical Care Centre (AMA) integrated
- UBS with an Eldery Heath Reference Unit (Unidade de referência de Saúde ao Idoso, URSI) integrated

1. What is the total number of the population that the UBS is responsible for? *

________________________________________________________________________

1. What is the total number of people enrolled in the UBS? *

________________________________________________________________________

1. What is the total number of active registered patients in the UBS? *

________________________________________________________________________

1. What is the total number of professionals in the UBS? (Fill in according to the example: 4 general practitioners, 1 paediatrician, 2 nurses, 1 nursing assistant. If your unit is mixed - AMA/UBS, AMA/ESF, UBS/ESF - please separate by the team of each format) *

_________________________________________________________________________

_________________________________________________________________________

_________________________________________________________________________

_________________________________________________________________________

_________________________________________________________________________

1. Does the UBS have working computers? If yes, how many are there? *

________________________________________________________________________

1. What software/programs does the UBS use? *

*Select all that apply.*

- E-SUS (Sistema Integrado de Saúde; Integrated Health System)
- SIGA (Sistema Integrado de Gestão; Integrated Management System)
- SIGA-PEP (Sistema Integrado de Gestão-Prontuário Eletrônico do Paciente; Integrated Management System)
- GSS (Gestão de Sistemas de Saúde; Health System Management)
- Other: ___________________________________________

**Local diagnosis on primary care / hypertension**

1. Does your unit know the total number of diagnosed hypertensive patients?*

*Mark only one*

- Yes
- No

1. Does your unit know the total number of registered hypertensive patients? *

*Mark only one.*

- Yes
- No

1. If yes, what is the number of registered hypertensive patients?

________________________________________________________________________

1. Does your unit have any strategy for recording the number and/or proportion of patients who control their blood pressure? *

*Mark only one*

- Yes
- No

1. Does your unit have a standard tool to collect information in the medical chart for the follow-up of hypertensive patients? *

*Mark only one.*

- Yes
- No

1. Does your unit have an information system to register hypertensive patients? *

*Mark only one.*

- Yes
- No

1. Does your unit have the number of hypertensive patients not followed-up in the last quarter?*

*Mark only one.*

- Yes
- No

1. Does your unit have the number of staff trained in the follow-up of hypertensive patients? If yes, list the category(ies) of trained professionals *

_________________________________________________________________________

_________________________________________________________________________

_________________________________________________________________________

_________________________________________________________________________

_________________________________________________________________________

1. Which protocol and/or guideline do the professionals follow to provide care for hypertensive patients? *

________________________________________________________________________

1. On average, what is the number of medical consultations with hypertensive patients per month? *

_________________________________________________________________________

_________________________________________________________________________

_________________________________________________________________________

_________________________________________________________________________

_________________________________________________________________________

1. On average, what is the total number of nursing consultations with hypertensive patients per month? *

________________________________________________________________________

1. On average, what is the total number of dental appointments with hypertensive patients per month? *

________________________________________________________________________

1. On average, what is the total number of pharmaceutical consultations with hypertensive patients per month? *

________________________________________________________________________

*Calculation of LDL (light-density lipoprotein) = Total cholesterol - HDL cholesterol - (triglycerides/5)

**Attendance to hypertensive patient**

1. Indicate which exams are requested during medical and nursing consultations *

*Select all that apply.*

- Electrocardiogram
- Total cholesterol test*
- Triglycerides test*
- Urine type 1
- Fasting blood glucose
- High-density lipoprotein (HDL) cholesterol test
- Creatinine test
- Potassium test
- Albuminuria/microalbuminuria

1. Doe the unit record and follow-up on annual re-evaluation exams requested by the physician? *

*Mark only one.*

- Yes
- No

1. What instructions are given to the patient before the blood pressure measurement? *

_________________________________________________________________________

_________________________________________________________________________

_________________________________________________________________________

_________________________________________________________________________

_________________________________________________________________________

1. When prescribing the amount of medication, does the nurse pay attention to the date of the next medical appointment, not exceeding 180 days (according to Ordinance No. 338/2014-SMS. G.) *

*Mark only one.*

- Yes
- No

1. How is the patient's blood pressure measured? *

_________________________________________________________________________

_________________________________________________________________________

_________________________________________________________________________

_________________________________________________________________________

_________________________________________________________________________

1. Tick which risk factor identification(s) method(s) professionals use on a daily basis

*Select all that apply*

- BMI calculation and classification
- Abdominal circumference
- Classification of hypertensive patients

1. How many times a year does the unit follow-up on low and medium risk patients? *

________________________________________________________________________

1. How many times a year does the unit follow-up on high-risk patients? *

________________________________________________________________________

1. In your unit, is there any strategy to improve the qualification of the individual medical record? Comment if possible. *

_________________________________________________________________________

_________________________________________________________________________

_________________________________________________________________________

_________________________________________________________________________

_________________________________________________________________________

**Promotion of quality of life**

1. In your unit, is there a medical record for the encouragement of a healthy lifestyle? Comment if possible. *

_________________________________________________________________________

_________________________________________________________________________

_________________________________________________________________________

_________________________________________________________________________

_________________________________________________________________________

1. Does your unit make information materials about hypertension available to patients? Comment if possible *

_________________________________________________________________________

_________________________________________________________________________

_________________________________________________________________________

_________________________________________________________________________

_________________________________________________________________________

1. Does your unit promote welfare activity(ies) for hypertensive patients? Comment if possible *

_________________________________________________________________________

_________________________________________________________________________

_________________________________________________________________________

_________________________________________________________________________

_________________________________________________________________________

1. How many activities were performed? *

________________________________________________________________________

1. How many participants are in the activity(ies)?*

________________________________________________________________________

1. The team is able to: *

*Select all that apply.*

- Motivate the patient to use the prescribed medications and lifestyle changes
- Inform about the importance of the correct use of medication
- Clarify the importance of taking daily doses of medication
- Involve caregivers and family members in the treatment (continuous user support)

1. Does the unit implement the Primary Care Access and Quality Improvement Program (Programa de Melhoria do Acesso e da Qualidade; PMAQ)? If yes, do you monitor the adult health/hypertension axis using the proposed instruments? *

________________________________________________________________________

1. Does the unit organize care according to the adult/hypertension care pathway? *

*Mark only one.*

- Yes
- No

1. Are referral and counter-referral guides commonly used? *

*Mark only one.*

- Yes
- No

1. In your unit, which criteria are used for referral to medium and high complexity? *

_________________________________________________________________________

_________________________________________________________________________

_________________________________________________________________________

_________________________________________________________________________

_________________________________________________________________________

1. Is there an access regulation process for these referrals? *

*Mark only one.*

- Yes
- No

1. Does the patient diagnosed with hypertension leave the unit with a scheduled referral? *

*Mark only one.*

- Yes
- No

1. Does the community health agent (CHA) sensitize family members to treatment adherence during home visits?

*Mark only one.*

- Yes
- No

1. Does the CHA look for missing patients?

*Mark only one.*

- Yes
- No

1. Does the CHA motivate the user to use the prescribed medications and lifestyle changes?

*Mark only one.*

- Yes
- No

1. Does the CHA have the knowledge necessary to inform about the importance of the correct use of medication?

*Mark only one.*

- Yes
- No

1. Does the CHA have the necessary knowledge to clarify the importance of taking/dosing daily medication?

*Mark only one.*

- Yes
- No

1. Does the CHA have the ability to involve the caregiver and family members in the treatment (continuous support of the user)?

*Mark only one.*

- Yes
- No

1. Does the unit perform territorialisation of its coverage area in order to identify the resources and equipment available in the region? *

*Mark only one.*

- Yes
- No
- Other: ___________________________________________
