# Supplementary material for: Situational analysis of hypertension management at primary health care level in São Paulo, Brazil: population, healthcare professional and health system perspectives
Source: BMC Health Serv Res. 2024 May 28;24:668. doi: 10.1186/s12913-024-10978-1 (PMC11134720; doi:10.1186/s12913-024-10978-1)
Supplement: Supplementary file 2 — Supplementary Material 2: Questionnaire used in the district of Penha. [file 12913_2024_10978_MOESM2_ESM.docx]

**Supplementary file 2.** Questionnaire used in the district of Penha.

Hi, we are the Better Hearts Better Cities Initiative team =)

The Municipal Health Secretariat (Secretaria Municipal de Saúde, SMS) of São Paulo is increasing its efforts to fight chronic non-communicable Diseases (NCDs) through the implementation of the protocol "Better Hearts Better Cities”. In this sense, the "Better Hearts Better Cities” initiative brings together national and international partners to support the SMS and accelerate this implementation.

The purpose of this questionnaire is to gather some information so that we can confirm data and get to know your unit better, so that the project can provide the most adequate support to the main needs and challenges of your primary healthcare unit (Unidade Básica de Saúde, UBS) regarding the line of care related to NCDs.

We suggest that it be answered as a team, since some questions will need confirmation from other people/areas.

Rest assured that this questionnaire is NOT an evaluation and does not intend to evaluate or expose each unit. It is the moment to share and point out structural and daily issues, as it will be fundamental for us to, together, create solutions that really make sense for everyone's reality.

We count on the information from your UBS and thank you in advance!

Better Hearts Better Cities Project Team - Tellus Institute

*To access more details about the project, access the site: http://www.cuidandodoseucoracao.org/

**Any questions, feel free to contact us via e-mail or by phone/Whatsapp: *contacts suppressed from this file*.

* Mandatory

1. E-mail *

_________________________________________________________________________

1. What is your name? *

_________________________________________________________________________

1. What is your position/function? *

*Mark only one.*

- Manager
- Nurse responsible technician (RT)
- Pharmacist
- Doctor
- Social assistant
- Administrative

Other: ___________________________________________

**General data of your unit**

1. To begin, select the name of your Health Unit: *

*Mark only one.*

- VALIDACAO - STS / SECONCI
- AMA/UBS Cangaíba - Doutor Carlos Gentille de Mello
- AMA/UBS Chácara Cruzeiro do Sul
- AMA/UBS Jardim Nordeste
- AMA/UBS Integrada Padre Manoel da Nóbrega
- AMA/UBS Vila Silvia
- UBS AE Carvalho
- UBS Dr. António Pires F. Villalobo
- UBS Patriarca - Hermenegildo Morbin Jr
- UBS Emilio Santiago
- UBS Engenheiro Goulart Doutor José Pires
- UBS Engenheiro Trindade
- UBS Jardim Maringá Vila Talarico
- UBS Jardim São Francisco
- UBS Jardim São Nicolau
- UBS Vila Aricanduva
- UBS Vila Esperança - Dr. Cássio Bittencourt Filho
- UBS Vila Granada
- UBS Vila Guilhermina
- UBS Vila Matilde
- UBS Padre José de Anchieta
- UBS Parque Arthur Alvim

1. Now tell us, what is the national registry of health establishments (Cadastro Nacional de Estabelecimentos de Saúde; CNES) number of the unit of the unit? *

_________________________________________________________________________

1. How many inhabitants does the coverage area of your UBS include? *

*Put the estimated population within the coverage area, not necessarily the number of people registered (we will ask this later).*

_________________________________________________________________________

1. From which source did you get this data? (number of inhabitants/people covered by the coverage area of the UBS) *

_________________________________________________________________________

1. What is the total number of registered and active patients/persons (with open and/or updated medical records in the last 5 years) in the UBS? *

_________________________________________________________________________

1. From what source did you get this data? (number of active registers in the UBS) *

_________________________________________________________________________

1. Considering the last 12 months, on average, what is the number of visits per month in the UBS? *

*If your unit is integrated, put here the UBS services only, and the AMA ones you can put in the next question.*

_________________________________________________________________________

1. From what source did you get this data? (number of visits per month in the UBS) *

_________________________________________________________________________

1. Considering the past 12 months, on average, what is the number of visits per month in the AMA? (If your facility does not have an AMA, skip the next section).

_________________________________________________________________________

1. From what source did you get this data? (number of visits to the AMA per month)?

_________________________________________________________________________

**Non-communicable disease care process management - Data, Sources and Tools**

1. How many blood pressure (BP) measurement procedures have been performed in the last 30 days? *

_________________________________________________________________________

1. From what source did you get this data? (number of BP measurement procedures) *

*Mark only one.*

- Primary care module (Modulo de Atenção Básica, MAB) (Integrated Health Care Management System in São Paulo; Sistema Integrado de Gestão de Assistência à Saúde de São Paulo, SIGA)
- Statistical data report (Quantity of procedures conducted per unit)
- Outpatient production bulletin (Boletim de Produção Ambulatorial, BPA)
- TABWIN
- TABNET
- e-SUS (Sistema Único de Saúde; unified health system)
- Others: ___________________________________________

1. Does your unit know (have records) of the number of diagnosed/identified hypertensive patients? *

*Mark only one.*

- Yes
- No

1. If "Yes", report the total number of diagnosed (identified/registered) hypertensive patients in your unit. (If "No", go on to the next questions)

*Consider the active subscribers from the last 5 years*

_________________________________________________________________________

1. From what source did you get this data (number of identified hypertensive patients/patients)? *

*Mark only one.*

- Report MAB (SIGA)
- Spreadsheet (Excel or similar) prepared by the UBS
- System (software) provided by the social organization
- TABWIN
- TABNET
- e-SUS
- Others: ___________________________________________

1. Which tool(s) does your UBS use to keep track of the patients diagnosed as hypertensive?*

*Mark only one per line.*

|  | Yes | No |
| --- | --- | --- |
| Spreadsheet (Excel or similar) prepared by the UBS itself |  |  |
| System (software) provided by the social organization |  |  |
| Individual attendance sheet (Ficha de Atendimento Individual, FAI) that feeds into SIGA |  |  |
| FORM-SUS |  |  |
| Other |  |  |

1. How many stratified hypertensive patients does the UBS have? *

*If not, enter the number 0 (zero)*

_________________________________________________________________________

1. Which tool(s) does the UBS use to keep track of the hypertensive patients who have been stratified? *

*Mark only one per line.*

|  | Yes | No |
| --- | --- | --- |
| Spreadsheet (Excel or similar) prepared by the UBS itself |  |  |
| System (software) provided by the social organization |  |  |
| FORM-SUS |  |  |
| Other |  |  |

1. What is the number of hypertensive patients seen in the last 30 days in the UBS?*

*If you don't have this number, type "0".*

_________________________________________________________________________

1. From what source did you get this data (hypertensive patients seen in the last 30 days in the UBS)? *

*If you don't have this number, type "0".*

_________________________________________________________________________

1. Can the unit somehow identify how many medical appointments with hypertensive patients were made per month?

*Mark only one.*

- Yes
- No
- Other: ___________________________________________

1. If "yes", what is the number of medical consultations performed in the last month with hypertensive patients?

_________________________________________________________________________

1. What is the number of nursing consultations performed in the last month with hypertensive patients? *

_________________________________________________________________________

1. From what source did you get this data (medical consultations and nursing consultations made by hypertensive patients)? *

_________________________________________________________________________

1. Does your unit perform pharmaceutical consultations? *

*Mark only one.*

- Yes
- No

1. If "Yes", what is the number of pharmaceutical consultations performed in the last month with hypertensive patients?

_________________________________________________________________________

1. From what source did you get this data? (pharmaceutical consultations made by hypertensive patients) *

_________________________________________________________________________

**Strategies and practices of the line of care – Chronic non-communicable diseases**

1. What protocol and/or guideline do professionals follow for the care of patients with hypertension?*

_________________________________________________________________________

1. Has the unit implemented the Primary Care Access and Quality Improvement Program (Programa de Melhoria do Acesso e da Qualidade, PMAQ)? *

*Mark only one.*

- Yes
- No

1. If yes, do you monitor the adult health/hypertension axis using the proposed instruments? Comment if possible. *

_________________________________________________________________________

_________________________________________________________________________

_________________________________________________________________________

_________________________________________________________________________

_________________________________________________________________________

1. Has your UBS instituted any of these strategies (have you set up, formalized, documented and/or signposted them in a place accessible to the team and/or patients of the UBS?)? *

*Mark only one per line.*

|  | Yes | No |
| --- | --- | --- |
| "Screening corner" (in a circulation area or room, with or without chair) set up to promote screening and care of chronic patients |  |  |
| Internal flow for referral when BP is altered |  |  |
| Strategy/process to ensure that people screened for abnormal BP in groups or active search actions make an appointment |  |  |
| Strategy/process/flow for follow-up of chronically ill patients that ensures subsequent consultations considering risk factors, hypertension stage and/or risk stratification |  |  |
| Specific strategy/process for active search for chronic uncontrolled patients or patients with poor adherence to treatment |  |  |
| Tool/sheet for monitoring controlled and uncontrolled chronic patients |  |  |
| Strategy/process for identifying cases of hypertensive crisis and/or need for removal |  |  |
| Flow of referral to secondary care |  |  |
| Timeline of permanent meetings for NCD with the team |  |  |

1. Has your UBS done any of the following actions in the last 30 days? *

*Mark only one per line.*

|  | Yes | No | I do not know |
| --- | --- | --- | --- |
| Internal active search actions in the UBS (in addition to the "Corner", another strategy to check BP of the patients of the UBS) |  |  |  |
| External active search actions (in the surroundings and coverage area) for tracking of people with altered BP and other risk factors |  |  |  |
| Internal action to promote health and information about NCDs to UBS patients (information session in the waiting room and at the UBS entrance, distribution of informative materials, preparation of materials to exhibit at the unit, etc.) |  |  |  |
| External actions to promote health with a focus on NCDs (informartion to the population, distribution of materials, participation in campaigns, etc.) |  |  |  |
| Activity groups and integrative complementary health practices (Práticas Integrativas e Complementares em Saúde, PICS) |  |  |  |
| Care group (specific for patients who have just been diagnosed with NCDs, according to the guideline of the Better Hearts Better Cities protocol) |  |  |  |
| Implemented the practice of elaborating “agreed self-care plans” (Planos de Autocuidado Pactuado, PAP) signed with the chronic patients, aiming to stimulate change in lifestyle (Mudança no Estilo de Vida, MEV) |  |  |  |
| Actions focused on the school health program (Programa Saúde na Escola, PSE) |  |  |  |
| Ensured keeping records of active search actions (internal and external), groups and groups activities, along with the number of participants, number of procedures performed (BP measured, blood glucose, etc.), and percentage of altered results |  |  |  |
| Ensured the scheduling of appointments for people who were either found to have to have alterations in BP during screening or who were identified to have risk factors during the active search activities |  |  |  |
| Updated the comparison between the number of chronic patients (hypertensive and diabetic) known by the UBS and the estimated prevalence (ISA Capital) |  |  |  |
| Held a meeting to discuss the protocol of Better Hearts Better Cities with the team |  |  |  |
| Provided the team with printouts of monthly lists of chronically ill patients to be followed (ensure follow-up, considering risk factors, stage of hypertension and/or risk stratification) |  |  |  |

1. What are the main factors that make it difficult/impractical for the unit to get referrals scheduled?

_________________________________________________________________________

_________________________________________________________________________

_________________________________________________________________________

_________________________________________________________________________

_________________________________________________________________________

**Training/continuing education in health**

1. Have the professionals in your unit received any training on the attention and care of chronic NCDs in the last year? *

*Mark only one.*

- Yes
- No
- I do not have this information

1. Could you tell us what this training was about (main topics covered)?

_________________________________________________________________________

39. Still on the training/capacity building in the context of NCDs, if it occurred, how many professionals of the current team were able to participate or received specific training in this area?

*If not, skip to the next section.*

_________________________________________________________________________

**Promotion, prevention and adherence support (lifestyle improvement)**

40. In your unit, do professionals provide written prescription for physical activity, diet, or other practices for lifestyle change? *

*Mark only one.*

- Yes
- No

41. If the answer is "Yes", comment if possible.

_________________________________________________________________________

_________________________________________________________________________

_________________________________________________________________________

_________________________________________________________________________

_________________________________________________________________________

42. Does the unit receive informative materials about NCDs (hypertension, diabetes...) to post in the UBS or make available to patients? *

*Mark only one.*

- Yes
- No

43. If "yes", how often does the unit receive informational materials about NCDs to post in the UBS or make available to patients? *

*Mark only one.*

- Every 3 months
- Every 6 months
- Once a year
- Every 2 years
- Every 3 months or more
- I do not recall receiving materials about non-cardiovascular diseases (NCDs)

44. Does the unit produce informational materials about NCDs to post or distribute to patients? *

*Mark only one.*

- Yes
- No

45. If "yes," how often does the unit produce informational materials about NCDs to post or distribute to patients? *

*Mark only one.*

- Every 3 months
- Every 6 months
- Once a year
- Every 2 years
- Every 3 months or more
- I do not recall receiving materials about NCDs

46. Does the unit promote group activities focused on hypertension? *

*Mark only one.*

- Yes
- No

47. How many group activities and integrative complementary health practices related to hypertension are currently taking place in the UBS? *

Examples: Physical activity groups, walking, stretching, dancing (zumba or other rhythms), anti-smoking, blood glucose self-monitoring program (AMG, programa de Automonotiramento Glicêmico), circular dance, yoga, tai chi, Lian Gong, Xian Gong, reiki, meditation...

_________________________________________________________________________

48. In total, how many people participated in these group activities in the last 30 days? *

*Please extract this data from SIGA.*

_________________________________________________________________________

49. Which of these activity groups, prevention and health promotion, and integrative complementary health practices (PICs) does the unit promote? *

*Mark only one per line.*

|  | Yes | No |
| --- | --- | --- |
| Physical activity group, walking, stretching |  |  |
| Dance group (zumba or other rhythmic activities) |  |  |
| Healthy eating/nutrition group |  |  |
| Anti-tobacco group |  |  |
| AMG Group |  |  |
| Circular dance |  |  |
| Yoga |  |  |
| Tai Chi |  |  |
| Lian Gong |  |  |
| Xian Gong |  |  |
| Reki |  |  |
| Meditation |  |  |
| Others |  |  |

50. With which organizations, schools, churches, businesses, public or private entities, has the UBS established some type of partnership for internal or external actions of promotion, prevention and/or screening in the last 12 months? *

*Please list the name of each (separated by commas).*

_________________________________________________________________________

_________________________________________________________________________

_________________________________________________________________________

_________________________________________________________________________

_________________________________________________________________________

51. Currently, what is the number of partners that are supporting the unit’s activities (internal or external)?

*Consider the number of institutions, schools, associations, commercial establishments, churches, or any other entity that the UBS has carried out a joint action with or received support from in the last 12 months.*

_________________________________________________________________________

52. Does the unit carry out campaigns in the territory? *

*Mark only one.*

- Yes
- No

53. What types of outreach events does the unit hold?

*Mark only one.*

|  | Yes | No |
| --- | --- | --- |
| Vaccination |  |  |
| BP testing |  |  |
| Glycaemia testing |  |  |
| Other lab exams |  |  |

54. Did the unit perform collective BP check-ups in the last months?? *

*Mark only one.*

- No
- Yes, within the last 30 days.
- Yes, in the last 3 months.
- Yes, within the last 6 months.
- Yes, in the last year.

55. If performed, report on the unit’s territorialization/analysis of its coverage area in order to identify the resources and equipment available in the region (for example: squares, bicycle paths, spaces for physical activities, gymnastics/stretching equipment, sports courts and other available spaces, schools and associations/NGOs, open marketplaces, etc.). *

*Mark only one.*

- Yes, and it is updated
- Yes, but it needs to be updated
- We still have not done it, but we intend to
- We did not do it

**Infrastructure and equipment**

56. Does the unit have printers available for printing routine materials? *

*Mark only one.*

- Yes
- No

57. Which of these systems does the UBS use? *

*Check all that apply.*

- E-SUS
- SIGA (Sistema Integrado de Gestão; Integrated Management System)
- SIGA-PEP (Sistema Integrado de Gestão-Prontuário Eletrônico do Paciente; Integrated Management System)
- GSS (Gestão de Sistemas de Saúde; Health System Management)
- Other: ___________________________________________

58. What other systems/software does the UBS use? *

_________________________________________________________________________

_________________________________________________________________________

_________________________________________________________________________

_________________________________________________________________________

_________________________________________________________________________

59. If your computers have Excel, what is the year/version of this software? *

_________________________________________________________________________

60. How many computers have this software? *

_________________________________________________________________________

61. Is the unit's electrocardiogram equipment working normally? *

*Mark only one.*

- Yes
- No

62. Does the unit have a room available for meetings, with chairs and seating for up to 30 people? *

*Mark only one.*

- Yes
- No

63. Is there a place near the unit that can be used for meetings/training, with chairs and space for up to 30 people (It can be a school, community center, public office, association/NGO, church, etc.)? *

*Mark only one.*

- Yes
- No

64. If yes, what is the name of this place?

*If no, skip to the next section.*

_________________________________________________________________________

_________________________________________________________________________

_________________________________________________________________________

_________________________________________________________________________

_________________________________________________________________________

**Contact with the unit**

65. What are the best days of the week and the most appropriate shifts of the day to carry out activities with the unit's staff? *

*Check all that apply.*

|  | Monday | Tuesday | Wednesday | Thursday | Friday | Saturday |
| --- | --- | --- | --- | --- | --- | --- |
| Morning |  |  |  |  |  |  |
| Afternoon |  |  |  |  |  |  |

66. What is the name of the UBS manager? *

_________________________________________________________________________

67. What is the cell phone number of the unit's manager?

_________________________________________________________________________

68. In the absence of the manager, who will be responsible for responding and making other referrals to the Initiative?

*Please put thename and function/category of the professional who will be the "focal point" for the dialogue with the project team.*

_________________________________________________________________________

69. What is the cell phone number of this professional who will be the "focal point" of the Initiative in the absence of the manager?

_________________________________________________________________________

THANKS! We will soon have the opportunity to personally visit each health unit and build with you the next steps of the Initiative.

In advance, we are very grateful for the attention and willingness of the whole team.

Best Regards,

"Better Hearts Better Cities” Team - Tellus Institute
