# Supplementary material for: Situational analysis of hypertension management at primary health care level in São Paulo, Brazil: population, healthcare professional and health system perspectives
Source: BMC Health Serv Res. 2024 May 28;24:668. doi: 10.1186/s12913-024-10978-1 (PMC11134720; doi:10.1186/s12913-024-10978-1)
Supplement: Supplementary file 3 — Supplementary Material 3: Shadowing of community health agent (CHA) form. [file 12913_2024_10978_MOESM3_ESM.docx]

**Supplementary file 3.** Shadowing of community health agent (CHA) form.

**Objective**: to identify influential stakeholders of the neighbourhood and the relationship between CHAs and patients.

**Questions asked to CHAs**

**Patients’ self-perception and communication with patients**

Do hypertensive people understand their condition and restrictions? Do they respect them? Are they afraid of the consequences?

What communication is done by the CHA concerning hypertension-focused and how?

Do patients understand the information being conveyed?

**Diet**

1. What is your understanding of maintaining a healthy life?
2. What do people in your region understand by healthy living? Are they conscious about being healthy?
3. How do people in your region eat? Can you describe a typical meal?
4. What are the central items in the pantry of a house in your region?
5. Who is “responsible” for the food preparation at home?
6. Where do people in your area usually shop?
7. Do people eat at home? Do they cook or buy ready-to-eat food?
   1. If they eat out: what restaurants/street food possibilities exist in the region?
8. Where do people eat out and where do you buy food to eat at home?
9. Someone who wants to be healthy, where does he/she eat and exercise?

**Physical activity**

1. Are people aware of the need for physical activity?
2. What physical activities take place here? Are there any walking, jogging, etc. groups?
3. How do people move within the neighbourhood?
4. What facilities are available for physical exercises?
5. What is the audience for each type of facility?
6. Are the available facilities used regularly?
7. Is obesity common?
8. Can you identify a type of physical activity that is more practiced by hypertensive patients (example: walking in the local garden, soccer at the community club)

**Others:** What are the main places with more movement/agglomeration of people?
